# Real-world impact of fremanezumab on migraine symptoms and resource utilization in the United States

**DOI:** 10.1186/s10194-021-01358-9

**Published:** 2021-12-20

**Authors:** Peter McAllister, Lois Lamerato, Lynda J. Krasenbaum, Joshua M. Cohen, Krishna Tangirala, Stephen Thompson, Maurice Driessen, Julian Casciano, Zenobia Dotiwala, Alexander Mauskop

**Affiliations:** 1 New England Institute for Neurology and Headache, New England Institute for Clinical Research and Ki Clinical Research, Yale University, 30 Buxton Farm Road, Suite 230, Stamford, CT 06905 USA; 2 Department of Public Health Sciences, Henry Ford Health System, Detroit, MI USA; 3 Teva Branded Pharmaceutical Products R&D, Inc., West Chester, PA USA; 4 Teva Branded Pharmaceuticals, Inc., Amsterdam, The Netherlands; 5 eMAX Health Systems, Delray Beach, FL USA; 6 Department of Neurology, SUNY Downstate Medical Center, New York Headache Center, New York, NY USA

**Keywords:** Migraine, Fremanezumab, Health care resource utilization, Migraine pain intensity, Headache frequency, Real-world efficacy

## Abstract

**Background:**

Fremanezumab, a fully humanized monoclonal antibody (IgG2Δa) that selectively targets calcitonin gene-related peptide (CGRP), is approved for migraine prevention in adults. Real-world data on the effectiveness of fremanezumab are limited. This retrospective, observational cohort study assessed patient-reported migraine symptoms, health care resource utilization (HCRU), and direct medical costs before and after fremanezumab treatment initiation.

**Methods:**

Data were extracted from September 2018 through June 2020 from the Midwest component of EMRClaims+®, an integrated health services database containing > 20 million medical records from national commercial insurance claims, Medicare claims, and regional electronic medical records. Patients included in the cohort analysis were aged ≥ 18 years and were administered fremanezumab, with enrollment or treatment history for ≥ 6 months prior (pre-index) to initiating fremanezumab (index date) and ≥ 1 month after the index date (post-index), and without pregnancy or pregnancy-related encounters during the study period. Patient-reported headache frequency, migraine pain intensity (MPI), composite migraine symptoms, and HCRU were assessed pre-index and ≥ 1 month after fremanezumab initiation. Wilcoxon signed-rank tests were used to compare means of migraine symptoms and outcomes and HCRU before and after fremanezumab initiation.

**Results:**

Overall, 172 patients were eligible for analysis. Of patients who self-reported (*n* = 129), 83.7% reported improvement in headache frequency or symptoms after fremanezumab treatment. Specifically, headache frequency decreased by 63% after fremanezumab initiation: mean (standard deviation) headache frequency was 22.24 (9.29) days per month pre-index versus 8.24 (7.42) days per month post-index (*P* < 0.0001). Mean MPI also decreased by 18% after fremanezumab initiation: MPI was 5.47 (3.19) pre-index versus 4.51 (3.34) post-index (*P* = 0.014). Mean emergency room (ER) visits per month decreased from 0.72 to 0.54 (*P* = 0.003), and mean outpatient visits per month decreased from 1.04 to 0.81 (*P* < 0.001). Mean hospitalizations per month decreased, but the results did not reach statistical significance (*P* = 0.095). Hospitalization and ER costs decreased, while outpatient costs increased, from pre-index to post-index, but differences were not statistically significant (*P* ≥ 0.232).

**Conclusions:**

Significant reductions in headache frequency, MPI, and HCRU were observed after fremanezumab initiation in patients with migraine in a US real-world setting.

## Background

Migraine is a complex neurologic disease that affects > 1 billion individuals worldwide, including 68.5 million in the United States and 80 million in Western Europe [1]. Individuals with migraine experience headache attacks lasting 4 to 72 h that are accompanied by a range of symptoms, which may include pulsing or throbbing headache pain, nausea, vomiting, photophobia, phonophobia, blurred vision, and aura [2, 3]. Experience of these symptoms commonly co-occurs with severe negative impact on daily activities and quality of life [1, 4], so much so that migraine is the second leading cause of years lived with disability worldwide [5]. In the United States, current disability-adjusted life-years for migraine are 2.4 million (95% uncertainty interval, 1.53–3.44 [1]). Additionally, health care resource utilization (HCRU), including primary health care visits, outpatient visits, and emergency room (ER) visits, is higher among patients with migraine compared to the general population [4]. Research demonstrates that HCRU is reduced among patients who experience more headache-free days [6]. Thus, migraine prevention is typically recommended in patients with attacks occurring ≥ 4 days per month to mitigate symptoms and associated disability [7, 8].

Fremanezumab is a fully humanized monoclonal antibody (IgG2Δa) that selectively targets calcitonin gene-related peptide (CGRP), a neuropeptide implicated in the pathophysiology of migraine [9, 10]. Fremanezumab has demonstrated efficacy, safety, and tolerability in adults with episodic migraine (EM; headache occurring < 15 days per month [11]) or chronic migraine (CM; ≥ 15 headache days per month, with attacks meeting migraine criteria ≥ 8 days per month over 3 months’ time [11]) in clinical trials [12, 13].

There are limited real-world effectiveness data for fremanezumab due to the recency (i.e., 2018) of its approval as a therapeutic indicated for migraine prevention in adults. Real-world effectiveness data are critical as they demonstrate the effects of a therapeutic when used under less structured conditions compared to clinical trials, including a broader patient population.

The lack of real-world data for fremanezumab is driven by the fact that (1) patient measures assessing information such as frequency of migraine attacks are not routinely captured, and (2) insurance claims or hospital encounter data are not appropriate data sources given the lack of structured symptom data. Quantifiable endpoints from real-world data are needed to provide evidence for fremanezumab’s effectiveness in less controlled, nonclinical trial conditions, which are considered in the development of coverage and utilization management policy. Thus, this retrospective, observational cohort study assessed patient-reported migraine symptoms and HCRU before and after fremanezumab treatment initiation.

## Methods

### Data source

Data were extracted from September 2018 through June 2020 from the Midwest component of EMRClaims+®, an integrated health services database containing > 20 million medical records from national commercial insurance claims, Medicare claims, and regional electronic medical records (EMR) collected from 1988 to the present, with over 3.1 million facility encounters added annually. Additionally, this database includes administrative insurance claims for approximately 690,000 individuals linked to an overlapping health care provider database of EMR data, including laboratory values and provider billing files. Standard longitudinal claims data, including pharmacy data and medical claims, are available for managed care members who have medical encounters within the EMR-reporting hospitals and outpatient facilities. Database elements are available as recently as 30 days from the time of extract, and EMR extracts are available as recently as the last 2 weeks from extract. Insured parties linked with the EMR are tracked through provider-aligned patient panels, managed care membership, and a Master Patient Index.

### Selection criteria for patient population

Patients were included in this cohort analysis if they were aged ≥ 18 years and administered fremanezumab, with enrollment or treatment history for ≥ 6 months prior (i.e., pre-index) to initiating fremanezumab treatment (index date) and ≥ 1 month after the index date (i.e., post-index), without pregnancy or pregnancy-related encounters during the study period. Patients concomitantly using other CGRP inhibitors were excluded from the study. The average follow-up duration after fremanezumab initiation for these patients was 12.8 months.

### Measures

Data were extracted on patient demographics such as age, gender, insurance payer, migraine diagnosis, related comorbidities, and ordering physician specialty. Data were also extracted on pre- and post-index patient-reported headache frequency, migraine pain intensity (MPI), patient-reported improvement in migraine symptoms, composite migraine symptoms, and all-cause HCRU (including inpatient hospitalizations, ER, and outpatient visits) and associated cost burden (based on billed charges) of each. Manual chart review was used to identify migraine characteristics.

Patient-reported improvement was recorded in the EMR by physicians and nurses, as a composite binary variable of “improvement” versus “no improvement” including patient-reported accounts of improvement (i.e., yes or no; irrespective of headache frequency) and patient-reported improvement in headache frequency. MPI was assessed via responses on a 10-point visual analog scale (VAS) [14] ranging from “no pain” to “worst pain” (0 = no pain, 10 = worst pain). To be conservative, the maximum reported headache frequency and intensity values at baseline and follow-up for each patient were used for the headache frequency and MPI.

### Statistical analysis

Descriptive statistics were computed to analyze sample demographics. Nonparametric Wilcoxon signed-rank tests, with a level of significance set at *P* < 0.05, were used to compare means of patient-reported headache frequency, MPI, and HCRU before and after fremanezumab initiation. To account for the differences in the follow-up duration for patients, mean HCRU measures were calculated per month.

## Results

Of the 330 patients with fremanezumab orders in the database, 127 patients were excluded because they did not receive administrations of this therapeutic, 26 due to lack of enrollment or treatment history ≥ 1 month after the index date, and 5 due to pregnancy diagnosis at any time during the study period. For the 172 patients who met all inclusion criteria and constitute the final sample, the mean (standard deviation [SD]) age was 46.0 (12.7) years; 84% (144/172) identified as female and 16% (27/172) as male. Fremanezumab was ordered by neurologists in 92% (158/172) of cases, with family medicine (5%; 8/172), internal medicine (2%; 3/172), pain medicine (< 1%; 1/172), and other (1%; 2/172) physicians placing the remaining orders. Patient demographics are displayed in Table 1.

**Table 1:** Demographics

Characteristic	All patients (***N*** = 172)
Gender, n (%)	
Female	144 (84)
Male	27 (16)
Age in years, mean (SD)	46.0 (12.7)
Mean follow-up in months, mean (SD)	12.8 (4.8)
Fremanezumab dosing, n (%)	
Monthly	168 (98)
Quarterly	3 (2)
Ordering physician, n (%)	
Neurology	158 (92)
Family medicine	8 (5)
Internal medicine	3 (2)
Pain medicine	1 (1)
Other	2 (1)

*SD* standard deviation

The most common migraine-related comorbid disease categories during the pre-index period included pain (37%; 63/172), psychiatric conditions (31%; 53/172), sleep disturbances (24%; 41/172), and cardiovascular comorbidities (22%; 38/172; Table 2). The most frequent comorbidities included insomnia (23%; 39/172), anxiety disorder (including generalized anxiety disorder; 21%; 36/172), chronic pain (21%; 36/172), back pain (19%; 33/172), depression (including major depressive disorder; 19%; 32/172), neck pain (16%; 28/172), and hypertension (16%; 28/172; Table 2).

**Table 2:** Patient comorbidities in the pre-index period

Comorbidities, n (%)	All patients (***N*** = 172)
Psychiatric disorders	53 (31)
Depression (including MDD)	32 (19)
Anxiety disorders (including GAD)	36 (21)
Depression and anxiety	19 (11)
Panic disorder	2 (1)
Bipolar spectrum disorders	8 (5)
Pain	63 (37)
Fibromyalgia	16 (9)
Chronic pain	36 (21)
Back pain	33 (19)
Neck pain	28 (16)
Sleep disorders	41 (24)
Insomnia	39 (23)
Restless leg syndrome	4 (2)
Sleep apnea	18 (10)
Digestive disorders	28 (16)
Irritable bowel syndrome	2 (1)
Constipation	9 (5)
Ulcer	20 (12)
Gastroesophageal reflux disease	19 (11)
Respiratory disorders	29 (17)
Allergies	10 (6)
Sinusitis	11 (6)
Bronchitis	2 (1)
Asthma	17 (10)
Cardiovascular disorders	38 (22)
Hypertension	28 (16)
High cholesterol	15 (9)
Stroke	1 (1)
Mitral valve prolapse	1 (1)
Postural orthostatic tachycardia syndrome	2 (1)
Neurologic disorders	8 (5)
Epilepsy	8 (5)
Autoimmune disorder	2 (1)
Rheumatoid arthritis	2 (1)
Hormonal disorder	1 (1)
Polycystic ovarian syndrome	1 (1)
Endocrine disorder	21 (12)
Diabetes	16 (9)
Hypothyroidism	7 (4)
Metabolic disorders	18 (10)
Obesity	18 (10)

*MDD* major depressive disorder, *GAD* generalized anxiety disorder

### Headache frequency and migraine pain intensity

**Fig. 1:**
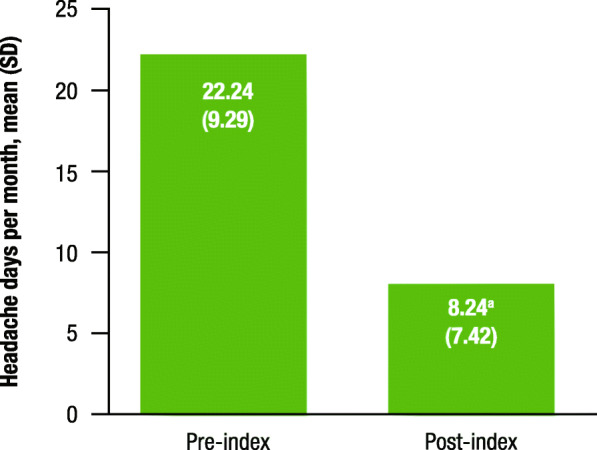
Change in headache frequency. SD, standard deviation. ^a^*P* < 0.001 for pre-index vs. post-index

Pre- and post-index headache frequency data were available for 76 patients; however, data on 22 of these patients were excluded from analysis because their self-reported “improvement” was inconsistent with headache frequency reports (*n* = 15) or their self-reported “no improvement” was inconsistent with headache frequency reports (*n* = 7). The 54 patients eligible for headache frequency analysis experienced a significant decrease in the mean (SD) number of headache days per month, from 22.24 (9.29) pre-index to 8.24 (7.42) post-index (*P* < 0.0001); a mean reduction of 14.00 days (63%; Table 3, Fig. 1).

**Table 3:** Patient-reported improvement, headache frequency, and migraine pain intensity

Parameter		***P*** value
Improvement, n (%)^a^	(*n* = 129)	
Yes	108 (84)	
No	21 (16)	
Headache frequency (overall)	(*n* = 54)	
Pre-index, mean (SD)	22.24 (9.29)	
Post-index, mean (SD)	8.24 (7.42)	
Difference in days, mean^b^	14.00	< 0.001
Difference %, mean	63%	
MPI (overall)	(*n* = 74)	
Pre-index, mean (SD)	5.47 (3.19)	
Post-index, mean (SD)	4.51 (3.34)	
Difference in days, mean^b^	0.96	0.014
Difference %, mean	18%	

*SD* standard deviation, *MPI* migraine pain intensity

^a^Not reported, *n* = 43

^b^Nonparametric Wilcoxon signed-rank tests, with a level of significance set at *P* < 0.05, were used to compare means of patient-reported headache frequency and MPI before and after fremanezumab initiation

Overall, data on improvement in headache frequency or symptoms were available for 129 patients, of whom 84% (108/129) reported improvement in headache frequency or symptoms after fremanezumab treatment and 16% (21/129) reported no improvement after treatment.

**Fig. 2:**
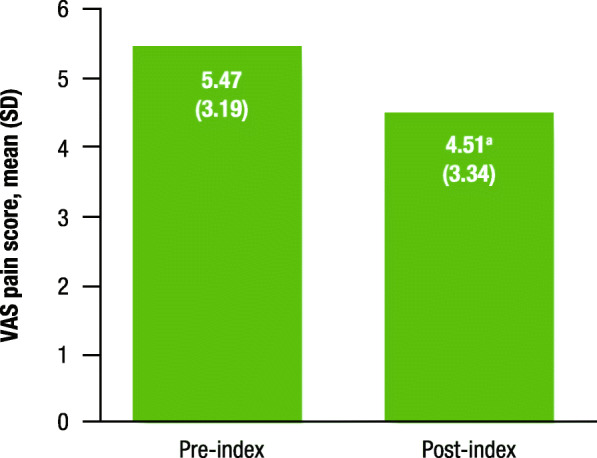
Change in the MPI VAS for headache. MPI, migraine pain intensity; VAS, visual analog scale; SD, standard deviation. ^a^*P* = 0.014 for pre-index vs. post-index

Pre- and post-index MPI was available for 74 patients. MPI decreased significantly by 18% after fremanezumab initiation, from a mean (SD) VAS pain score of 5.47 (3.19) pre-index to 4.51 (3.34) post-index (*P* = 0.014; Table 3, Fig. 2).

### Health care resource utilization

The percentage of patients with any inpatient hospitalizations (12% [20/172] vs. 10% [18/172]), any ER visits (50% [86/172] vs. 50% [86/172]), and any outpatient visits (94% [161/172] vs. 94% [162/172]) remained stable between the pre- and post-index periods. Mean (SD) inpatient hospitalizations per month decreased from 0.21 (0.79) pre-index to 0.16 (0.60) post-index; however, this decrease was not statistically significant (*P* = 0.095). Mean (SD) inpatient hospitalization cost per month also decreased from $2051.91 ($10,003.23) pre-index to $1359.88 ($6261.16) post-index; similarly, this decrease was not statistically significant (*P* = 0.232; Table 4).

**Table 4:** Health care resource utilization

HCRU, mean (SD)	All patients (***N*** = 172)	Max	***P*** value^**a**^
Mean hospitalizations (per month)			
Pre-index	0.21 (0.79)	6.00	
Post-index	0.16 (0.60)	4.38	0.095
Mean hospitalization cost (per month)			
Pre-index	$2051.91 (10,003.23)	$93,688.35	
Post-index	$1359.88 (6261.61)	$55,951.04	0.232
Mean ER visits (per month)			
Pre-index	0.72 (1.26)	7.50	
Post-index	0.54 (0.89)	5.36	0.003
Mean ER visit costs (per month)			
Pre-index	$1300.89 (4377.38)	$47,351.24	
Post-index	$1258.30 (5966.90)	$73,758.67	0.825
Mean outpatient visits (per month)			
Pre-index	1.04 (1.19)	7.50	
Post-index	0.81 (0.87)	5.36	< 0.001
Mean outpatient visit costs (per month)			
Pre-index	$531.21 (1067.18)	$6246.26	
Post-index	$697.63 (1665.83)	$14,182.75	0.257
Mean outpatient visit costs (per month) without outliers (i.e., restricting data to cost ≤$5000.00)			
Pre-index	$465.96 (898.96)	$4813.15	
Post-index	$459.14 (823.01)	$4477.17	0.935

*HCRU* health care resource utilization, *SD* standard deviation, *ER* emergency room

^a^Nonparametric Wilcoxon signed-rank tests, with a level of significance set at *P* < 0.05, were used to compare means of HCRU measures before and after fremanezumab initiation

Mean (SD) ER visits per month decreased significantly from 0.72 (1.26) pre-index to 0.54 (0.89) post-index (*P* = 0.003); however, the decrease in mean (SD) ER visit costs per month, from $1300.89 ($4377.38) pre-index to $1258.30 ($5966.90) post-index, was not statistically significant (*P* = 0.825; Table 4).

Mean (SD) outpatient visits per month decreased significantly from 1.04 (1.19) pre-index to 0.81 (0.87) post-index (*P* < 0.001); however, mean (SD) outpatient visit cost per month increased from $531.21 ($1067.18) pre-index to $697.63 ($1665.83) post-index, but the change was not statistically significant (*P* = 0.257). An additional analysis with outpatient cost outliers (i.e., cost >$5000.00) data excluded yielded a decrease in mean (SD) outpatient visit cost per month from $465.96 ($898.96) pre-index to $459.14 ($823.01) post-index; however, this change was also not statistically significant (*P* = 0.935; Table 4).

## Discussion

This retrospective, observational cohort study demonstrated the real-world benefits of fremanezumab as a preventive treatment for migraine in adults. The results showed that fremanezumab treatment is associated with improvements in patient-reported headache frequency, MPI, and reductions in HCRU and costs. Overall, 84% of patients with available self-reported data in this study reported decreases in headache frequency or symptoms after treatment initiation. Regarding headache frequency, fremanezumab treatment resulted in a significant 14-day (−63%) decrease in the patient-reported average number of headache days per month. The 14-day (or −63%) decrease in the patient-reported average number of headache days per month observed in the present study adds to the body of evidence suggesting a significant clinical response to this therapeutic [13, 15, 16]. Of note, despite the shorter follow-up duration, the reduction in headache frequency observed in this real-world study was greater than those evidenced in clinical trials (follow-up duration, 3 to 6 months; −2.6 to −4.6 headache days per month) [12, 13, 17]. Further, a significant post-treatment decrease in the patient-reported average MPI of 18% was also observed. Taken together, these findings further speak to fremanezumab’s potential to mitigate the significant disability associated with migraine [13], which is the top cause of years lived with disability among those 15 to 49 years of age [18].

Previous real-world studies have demonstrated that CGRP pathway–targeted therapies are effective as preventive treatments for migraine in adults [19–22]. In a Spanish prospective observational study of 155 migraine patients with ≥ 8 headache days per month and ≥ 3 prior preventive medication failures, treatment with erenumab (*n* = 109) or galcanezumab (*n* = 46) for 3 months resulted in a ≥ 50% reduction in migraine days per month in 51.6% of patients (mean [SD] reduction in migraine days per month compared with baseline was −8.5 [7.7]) [20]. In a sample of 81 Italian patients with high-frequency EM and CM, treatment with galcanezumab for 3 months resulted in a significant decrease in monthly migraine days compared with baseline (EM, −8.5; CM, −11.5; both *P* < 0.0001) [21]. A multicenter retrospective chart review of US headache centers evaluated data from 1034 patients with CM [19]. Results showed that treatment with erenumab for ≥ 3 months resulted in a ≥ 50% reduction in mean headache/migraine days per month among 35% of patients. Further, 45% of patients reported improvement in physician-reported migraine severity, and average monthly outpatient visits decreased from 0.43 at baseline to 0.30 after erenumab initiation. A separate retrospective real-world analysis that evaluated 6-month follow-up data after ≥ 1 erenumab injection found that, for 43 patients with available data, monthly migraine days significantly decreased by 8.4 days from baseline [22]. In the current real-world study, fremanezumab treatment was associated with a 14-day reduction in the average number of headache days per month from baseline. Any cross-study comparisons among these real-world studies should be considered with caution given the differences in the follow-up periods, outcomes assessed, patient populations, disease severity, and insurance reimbursement policies by region.

Fremanezumab treatment was also associated with significant decreases in HCRU. Compared to pre-treatment, statistically significant post-treatment decreases in average ER (25%) and outpatient (22%) visits were observed. Decreases in average ER and outpatient visit costs per month were also observed; however, these changes were not statistically significant. While average inpatient hospitalizations and average inpatient hospitalization cost per month also decreased, the results did not reach statistical significance. Mean outpatient costs increased nonsignificantly. This finding was likely attributable to outliers; an additional analysis excluding outliers (outpatient cost >$5000.00) showed a decrease in mean outpatient costs, but the results remained nonstatistically significant. Overall, fremanezumab treatment was associated with reductions in HCRU, specifically, average ER and outpatient visits; however, the cost differences across HCRU were not statistically significant. Reductions in HCRU are pertinent factors in decision making for policy makers and payers, regarding reimbursement and medication coverage. For patients, reducing costs alone may alleviate the financial burden previously imposed by their disease.

A strength of this observational study is its novel examination of both clinical outcomes and HCRU and associated costs simultaneously, compared with most studies in the literature that evaluated one or the other. This study design allowed us to examine the association between improvements in clinical outcomes with fremanezumab treatment and HCRU and cost reductions. Another strength of this study is the methodology used to collect data. Specifically, the use of the large database allowed for the analysis of real-world data to demonstrate the benefits of fremanezumab over a longer, more recent timeframe. Additionally, the database is an independent data source, providing impartiality of data reporting and reducing the likelihood of selection bias. A limitation of this study was the loss of data from patients with discrepancies between self-reported improvement as a dichotomous “yes/no” measure from their actual headache frequency reported pre- and post-fremanezumab initiation. Although this resulted in data loss, it ensured validity between the 2 measures of improvement. Furthermore, the results were still statistically significant in an analysis that included all 76 patients with pre- and post-index headache frequency data. Some patients did not return for follow-up visits due to improvement in their migraine or other reasons, and some outcomes were reported by patients via telephone rather than directly in a clinical setting; both may have increased the potential for misreporting and bias. Further, this study did not measure or control for the potential confounding effects of other migraine preventive medications. The loss of data due to the exclusion of patients or patients who were lost to follow-up may have limited the potential to detect differences between the pre- and post-index periods. Further, the loss of data for patients who did not return for follow-up visits, potentially due to improvement of their migraine, may have reduced the effect size for post-index outcomes with fremanezumab. These limitations may potentially reduce the generalizability of these data for the overall population with migraine. Future studies may benefit from first conducting power analyses to determine the number of patients necessary in order to detect a significant effect for improvement.

This study highlights consideration for future studies aiming to further elucidate fremanezumab’s real-world effectiveness. Such studies should include a larger, more diverse sample to increase the generalizability of findings. Given the degree of comorbidities among patients in this sample, future studies may also use patients’ treatment history and associated diagnostic codes to control for the confounding effects of other medications (migraine or otherwise) and non–migraine-related encounters. Such factors may explain why outpatient visit costs were observed to increase in this study for some patients, while all other costs decreased. Finally, in addition to continuing to evaluate the safety and effectiveness of this therapeutic as a preventive treatment for migraine, future studies may include pre- and post- measures of days of work, productivity, and/or income to further assess the improvements in migraine-related disability associated with fremanezumab treatment.

## Conclusion

Fremanezumab treatment was associated with significant reductions in headache frequency, MPI, and HCRU, which may also improve quality of life for patients with migraine. Less HCRU translated to substantial reductions in hospitalization and ER costs. Fremanezumab treatment was also associated with patient-reported improvements in migraine-associated symptoms, which may be even more pervasive and burdensome than the headaches themselves. The data from this study may aid clinical decision making for patients with migraine and the development of coverage and utilization management policy.

## Data Availability

Anonymized data, as described in this manuscript, will be shared upon request from any qualified investigator by the author investigators or Teva Pharmaceutical Industries, Ltd.

## Abbreviations

CGRP: Calcitonin gene-related peptide
HCRU: Health care resource utilization
CM: Chronic migraine
EM: Episodic migraine
EMR: Electronic medical records
MPI: Migraine pain intensity
VAS: Visual analog scale
ER: Emergency room
SD: Standard deviation

## References

[1] GBD 2016 Headache Collaborators (2018) Global, regional, and national burden of migraine and tension-type headache, 1990–2016: a systematic analysis for the Global Burden of Disease Study 2016. Lancet Neurol 17:954–976 10.1016/S1474-4422(18)30322-3 PMC6191530 30353868

[2] Buse DC, Loder EW, Gorman JA, Stewart WF, Reed ML, Fanning KM (2013). Sex differences in the prevalence, symptoms, and associated features of migraine, probable migraine and other severe headache: results of the American Migraine Prevalence and Prevention (AMPP) study. Headache..

[3] Goadsby PJ, Holland PR, Martins-Oliveira M, Hoffmann J, Schankin C, Akerman S (2017). Pathophysiology of migraine: a disorder of sensory processing. Physiol Rev.

[4] Vo P, Paris N, Bilitou A, Valena T, Fang J, Naujoks C et al (2018) Burden of migraine in Europe using self-reported digital diary data from the Migraine Buddy^©^ application. Neurol Ther 7:321–332 10.1007/s40120-018-0113-0 PMC6283800 30293098

[5] GBD 2016 Disease and Injury Incidence and Prevalence Collaborators (2017). Global, regional, and national incidence, prevalence, and years lived with disability for 328 diseases and injuries for 195 countries, 1990–2016: a systematic analysis for the Global Burden of Disease Study 2016. Lancet.

[6] Doane MJ, Gupta S, Vo P, Laflamme AK, Fang J (2019). Associations between headache-free days and patient-reported outcomes among migraine patients: a cross-sectional analysis of survey data in Europe. Pain Ther.

[7] Ha H, Gonzalez A (2019). Migraine headache prophylaxis. Am Fam Physician.

[8] Charles A (2017). Migraine. N Engl J Med.

[9] Bigal ME, Walter S, Rapoport AM (2013). Calcitonin gene-related peptide (CGRP) and migraine current understanding and state of development. Headache.

[10] Scuteri D, Adornetto A, Rombola L, Naturale MD, Morrone LA, Bagetta G (2019). New trends in migraine pharmacology: targeting calcitonin gene-related peptide (CGRP) with monoclonal antibodies. Front Pharmacol.

[11] Arnold M (2018) Headache Classification Committee of the International Headache Society (IHS). The International Classification of Headache Disorders, 3rd edition. Cephalalgia 38:1–211 10.1177/0333102417738202 29368949

[12] Silberstein SD, Dodick DW, Bigal ME, Yeung PP, Goadsby PJ, Blankenbiller T (2017). Fremanezumab for the preventive treatment of chronic migraine. N Engl J Med.

[13] Dodick DW, Silberstein SD, Bigal ME, Yeung PP, Goadsby PJ, Blankenbiller T (2018). Effect of fremanezumab compared with placebo for prevention of episodic migraine: a randomized clinical trial. JAMA.

[14] Aicher B, Peil H, Peil B, Diener HC (2012). Pain measurement: visual analogue scale (VAS) and verbal rating scale (VRS) in clinical trials with OTC analgesics in headache. Cephalalgia.

[15] Blumenfeld AM, Bloudek LM, Becker WJ, Buse DC, Varon SF, Maglinte GA et al (2013) Patterns of use and reasons for discontinuation of prophylactic medications for episodic migraine and chronic migraine: results from the second International Burden of Migraine Study (IBMS-II). Headache 53:644–655 10.1111/head.12055 23458496

[16] Silberstein SD, Cohen JM, Yeung PP (2019). Fremanezumab for the preventive treatment of migraine. Expert Opin Biol Ther.

[17] Bigal ME, Edvinsson L, Rapoport AM, Lipton RB, Spierings EL, Diener HC (2015). Safety, tolerability, and efficacy of TEV-48125 for preventive treatment of chronic migraine: a multicentre, randomised, double-blind, placebo-controlled, phase 2b study. Lancet Neurol.

[18] Steiner TJ, Stovner LJ, Vos T, Jensen R, Katsarava Z (2018). Migraine is first cause of disability in under 50s: will health politicians now take notice?. J Headache Pain.

[19] Faust E, Pivneva I, Yang K, Betts KA, Ahmed Z, Joshi S (2021). Real-world treatment profiles, clinical outcomes, and healthcare resource utilization of patients with migraine prescribed erenumab: a multicenter chart-review study of US headache centers. Neurol Ther.

[20] Torres-Ferrus M, Gallardo VJ, Alpuente A, Caronna E, Gine-Cipres E, Pozo-Rosich P (2021) The impact of anti-CGRP monoclonal antibodies in resistant migraine patients: a real-world evidence observational study. J Neurol 268:3789–3798 10.1007/s00415-021-10523-8 33772636

[21] Vernieri F, Altamura C, Aurilia C, Brunelli N, Egeo G, Fofi L (2020). Effectiveness, safety, and tolerability of galcanezumab in a real-life setting in patients with migraine in Italy (the GARLIT study). Neurol Sci.

[22] Robblee J, Devick KL, Mendez N, Potter J, Slonaker J, Starling AJ (2020). Real-world patient experience with erenumab for the preventive treatment of migraine. Headache.

